# High Antigen Dose Is Detrimental to Post-Exposure Vaccine Protection against Tuberculosis

**DOI:** 10.3389/fimmu.2017.01973

**Published:** 2018-01-15

**Authors:** Rolf Billeskov, Thomas Lindenstrøm, Joshua Woodworth, Cristina Vilaplana, Pere-Joan Cardona, Joseph P. Cassidy, Rasmus Mortensen, Else Marie Agger, Peter Andersen

**Affiliations:** ^1^Department of Infectious Disease Immunology, Statens Serum Institut, Copenhagen, Denmark; ^2^Unitat de Tuberculosi Experimental, Institut per a la Investigació en Ciències de la Salut Germans Trias I Pujol, CIBER Enfermedades Respiratorias, Universitat Autònoma de Barcelona, Badalona, Barcelona, Spain; ^3^Veterinary Sciences Centre, School of Veterinary Medicine, University College Dublin, Belfield, Dublin, Ireland

**Keywords:** tuberculosis, post-exposure vaccination, vaccine dose, T cell quality, functional avidity, CAF01, H56, adjuvant

## Abstract

*Mycobacterium tuberculosis* (Mtb), the etiologic agent of tuberculosis (TB), causes 1.8M deaths annually. The current vaccine, BCG, has failed to eradicate TB leaving 25% of the world’s population with latent Mtb infection (LTBI), and 5–10% of these people will reactivate and develop active TB. An efficient therapeutic vaccine targeting LTBI could have an enormous impact on global TB incidence, and could be an important aid in fighting multidrug resistance, which is increasing globally. Here we show in a mouse model using the H56 (Ag85B-ESAT-6-Rv2660) TB vaccine candidate that post-exposure, but not preventive, vaccine protection requires low vaccine antigen doses for optimal protection. Loss of protection from high dose post-exposure vaccination was not associated with a loss of overall vaccine response magnitude, but rather with greater differentiation and lower functional avidity of vaccine-specific CD4 T cells. High vaccine antigen dose also led to a decreased ability of vaccine-specific CD4 T cells to home into the Mtb-infected lung parenchyma, a recently discovered important feature of T cell protection in mice. These results underscore the importance of T cell quality rather than magnitude in TB-vaccine protection, and the significant role that antigen dosing plays in vaccine-mediated protection.

## Introduction

One fourth of the world’s population is estimated to harbor latent *Mycobacterium tuberculosis* (Mtb) infection (LTBI), of which 5–10% will eventually develop active and transmittable tuberculosis (TB) (1, 2). This constitutes an enormous reservoir of potential TB disease, and developing a vaccine that can prevent reactivation in LTBI individuals would greatly impact the global TB burden (3). Furthermore, efficient therapeutic vaccination would be an essential tool in combating MDR/XDR-TB cases with low responsiveness to second-line antibiotics. However, it has proven very difficult to achieve vaccine protection in post-exposure or therapeutic animal TB models (4, 5), and little is known about the mechanisms of protection in the existing studies (6–8).

Evidence suggests a protective immune response against infection with Mtb is derived mainly from IFN-γ-producing Th1 cells that activate infected macrophages, since CD4-deficient, IFN-γ- and inducible nitric oxide synthase (iNOS)-KO mice are highly susceptible to Mtb infection compared to wild-type strains (9–12). However, the last 10–20 years of research has shown that TB immunity is not as straightforward as previously understood, with some studies even suggesting that classical Th1-derived cytokines are not necessary for protection (13, 14).

Recently, it was shown that optimal protective capacity of T cells against Mtb infection relies on the ability of T cells to home into the lung parenchyma to make close contact with granuloma-resident Mtb-infected host cells (15–18). Notably, vaccination of naïve mice with H56/CAF01 in a preventive mouse model of TB induced high numbers of protective CD4 T cells with these homing attributes (16).

The goal of the current study was to examine in greater detail the mechanisms behind protection of H56 (Ag85B-ESAT-6-Rv2660c) formulated in CAF01 in a post-exposure model of TB, in which T cells have already been primed by Mtb prior to vaccination, resulting in a more terminally differentiated and less protective phenotype compared to H56/CAF01 vaccination of naïve animals (16). In preventive models, we have shown that the induction and retention of central memory (Tcm)-like T cells co-producing IL-2 and TNF are essential for long-term protection (19–22), and furthermore that vaccine antigen dose significantly affects T cell functional avidity (23), differentiation status, and their subsequent protection against TB (24). In line with this, recent human data from dose-escalating studies with H56 and related protein hybrids have shown that vaccination with higher doses of antigen results in a higher degree of T cell differentiation. Importantly, this phenomenon is accentuated in Quantiferon (QFT) positive individuals (24–28). In mice, the biological relevance of these findings is underscored by multiple groups observing inferior TB protection of terminally differentiated CD4 T cells characterized by high KLRG1 expression with a decreased ability to home into the lung parenchyma in adoptive transfer studies (15, 29, 30).

In the present paper, we use the standardized mouse model of pre- and post-exposure vaccination with H56/CAF01 to demonstrate that post-exposure protection against TB is very sensitive to vaccine antigen dose, in contrast to the preventive setting. We find that higher vaccine antigen doses drive terminal differentiation, decreased functional avidity, and resulted in a non-protective state of vaccine-promoted T cells with a decreased ability to home into the lung parenchyma. We conclude that T cells in the context of an Mtb primed immune response are highly susceptible to terminal differentiation by excess stimulation of high vaccine doses.

## Materials and Methods

### Animal Handling

Studies were performed with 6- to 8-week-old ♀CB6F1 mice (♂C57BL/6x♀Balb/c; Envigo, Denmark). Mice were housed in appropriate animal facilities at Statens Serum Institut, and experiments conducted in accordance with regulations of the Danish Ministry of Justice and animal protection committees by Danish Animal Experiments Inspectorate Permit 2009/561-1655, 2012-15-2934-00272, 2014-15-2934-01065 and in compliance with EU Directive 2010/63 and the U.S. Association for Laboratory Animal Care recommendations for the care and use of laboratory animals. Animals were rested 1 week after arrival and before commencement of experiments.

### Bacteria

*M. tuberculosis* Erdman were grown at 37°C on Middlebrook 7H11 (BD Pharmingen) agar or in suspension in Sauton medium (BD Pharmingen) as previously described (31).

### Antigens and Vaccines

Production of the H56 fusion protein has been described previously (6). For restimulation of cell cultures, 18-mer peptides covering the antigen-sequence with 10 a.a. overlaps (2 µg/ml per peptide) or recombinant proteins (Ag85B and H56; 5 µg/ml) proteins produced in *E. coli* were used.

### Experimental Infections and the Preventive and Post-Exposure TB Models

The preventive TB model has been previously described (6). Briefly, naïve mice were immunized three times with the indicated doses of H56 in CAF01, and challenged with a low dose of 10–50 CFU virulent Mtb Erdman with an inhalation exposure chamber (Biaera-AeroMP, capacity = 80 mice per aerosol round). Mice from different aerosol rounds were randomized to take potential aerosol round variation into consideration. Six weeks after challenge, mice were euthanized, and numeration of bacterial counts (CFUs) in the lungs were determined by serial threefold dilutions of whole-organ homogenates on 7H11 medium and presented as log10 means of bacterial counts. The post-exposure model was previously described (6, 7). Briefly, mice were challenged by the aerosol route with ~10–50 CFU of Mtb Erdman/mouse (Glas-Col/Biaera-AeroMP, capacity = 30/80 mice per aerosol round, respectively) and randomized if necessary as described above. Six weeks after the infection, mice were subjected to an antibiotic chemotherapy treatment (100 mg/l rifabutin, 100 mg/l isoniazid) in drinking water from week 6 to 12 of infection. Mice were immunized three times 3 weeks apart starting in the second last week of chemotherapy (weeks 10, 13, and 16 post challenge; Figure S1A in Supplementary Material). Bacterial counts after therapy was undetectable in the lungs (detection limit 10 CFU), and protection was assessed by bacterial enumeration in lungs 25 weeks after treatment (week 37 after infection).

### Immunizations

Mice were immunized three times at 2-week (preventive) or 3-week intervals (post-exposure) s.c. with a range of doses (0.005–50 µg) of recombinant H56 formulated in CAF01 as described (19).

### Cell Cultures and Immunological Readouts

**Figure 1:**
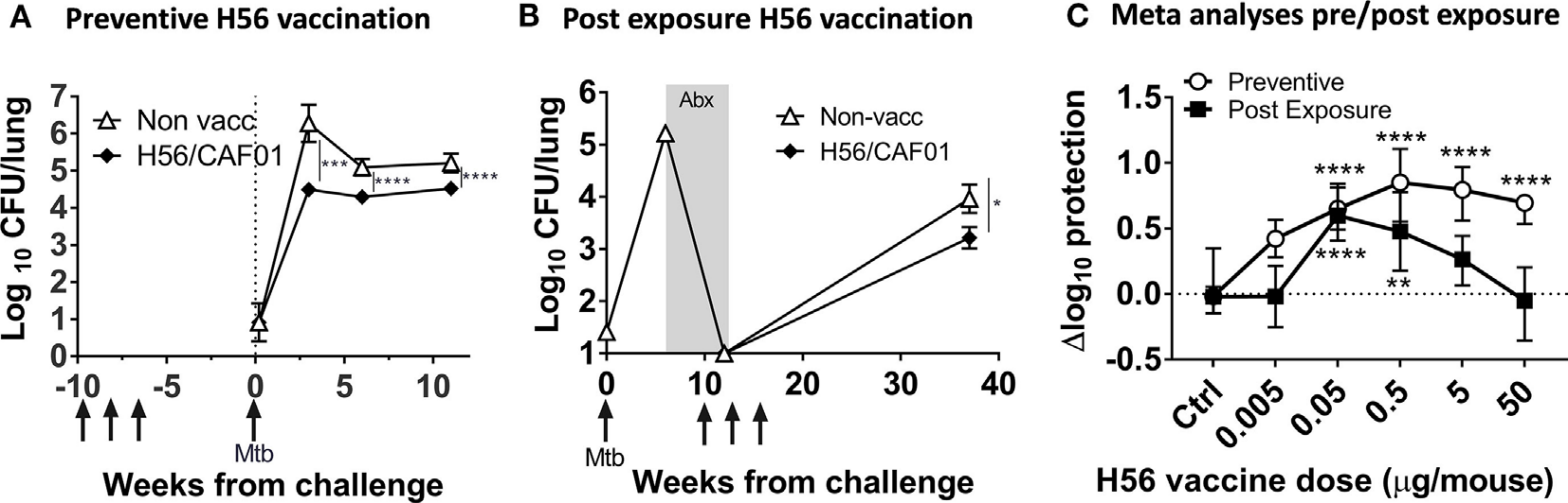
High dose H56 in post-exposure vaccination is detrimental to protection against *Mycobacterium tuberculosis (*Mtb). Total lung bacterial burden (log_10_ CFU) for unvaccinated control (open triangle) and 5 µg H56 in CAF01 vaccinated (closed diamond) mice in preventive **(A)** and post-exposure **(B)** vaccination TB protection models. **(A)** CB6F1 mice were vaccinated (gray arrows) s.c. three times and rested 6 weeks before low dose aerosol Mtb challenge and subsequent lung CFU determination at weeks 3, 6, and 12 post-infection. Symbols represent mean ± SEM of *n* = 6–8 mice/group. ***p < 0.001, ****p < 0.0001 by two-way ANOVA with Tukey’s posttest for multiple comparisons at each time point. **(B)** CB6F1 mice were aerosol-infected with Mtb and treated with antibiotics (gray box; Abx) prior to three s.c. H56 vaccinations (gray arrows, weeks 10, 13, and 16) and subsequent CFU determination 37 weeks post-infection. Symbols represent mean ± SEM, *n* = 4–6 mice/grp (weeks 0–12) and 15/group (week 37). *p < 0.05 Mann–Whitney test at week 37. **(C)** Meta-analysis of Δlog_10_ protection values (see Materials and Methods for calculation) for the indicated H56 dose using combined data from three independent preventive (open circles, 6 weeks post Mtb) and nine independent post-exposure (filled squares, 37 weeks post Mtb) vaccination experiments. Symbols, median ± 95% CI of *n* = 21–129 mice/group. **p < 0.01, ****p < 0.0001 by Kruskall–Wallis non-parametric test with Dunn’s test for multiple comparisons (vaccine groups vs. controls, separate analyses for preventive/post-exposure model).

Spleen and lung lymphocytes were isolated at different time points after either vaccination and infection and stimulated *in vitro* for cytokine release analysis by intracellular cytokine stain (ICS) or ELISA of culture supernatants as previously described (20). In the flow cytometric analysis of T cell magnitude and polyfunctionality, we showed data from seven experiments: five of the nine individual experiments shown in Figure 1C in which identical analyses of pulmonary immune responses were assessed by ICS and flow cytometry 1 week after last vaccination, and that contained both 0.05 and 50 µg H56 doses within the same experiments (Exp. 1, 4, 6, 7, 9 in Figure S1B in Supplementary Material); and two additional experiments in which only immune responses at week 17, but no long-term protection at week 37, was assessed, to include a total of *n* = 21 mice/group.

### Normalization of CFU to Compare Protection

As previously described (7), we assessed the delta log_10_ protection by subtracting individual log10 CFUs from the average log_10_ CFU of the control group:

Log protection = Average log_10_ CFU (unvaccinated control group) − individual log_10_ CFU.

Medians were used in Figure 1C since not all post-exposure experimental log_10_ CFUs were normally distributed. Similar results were obtained using means and medians.

### Histopathological Assessment

**Figure 2:**
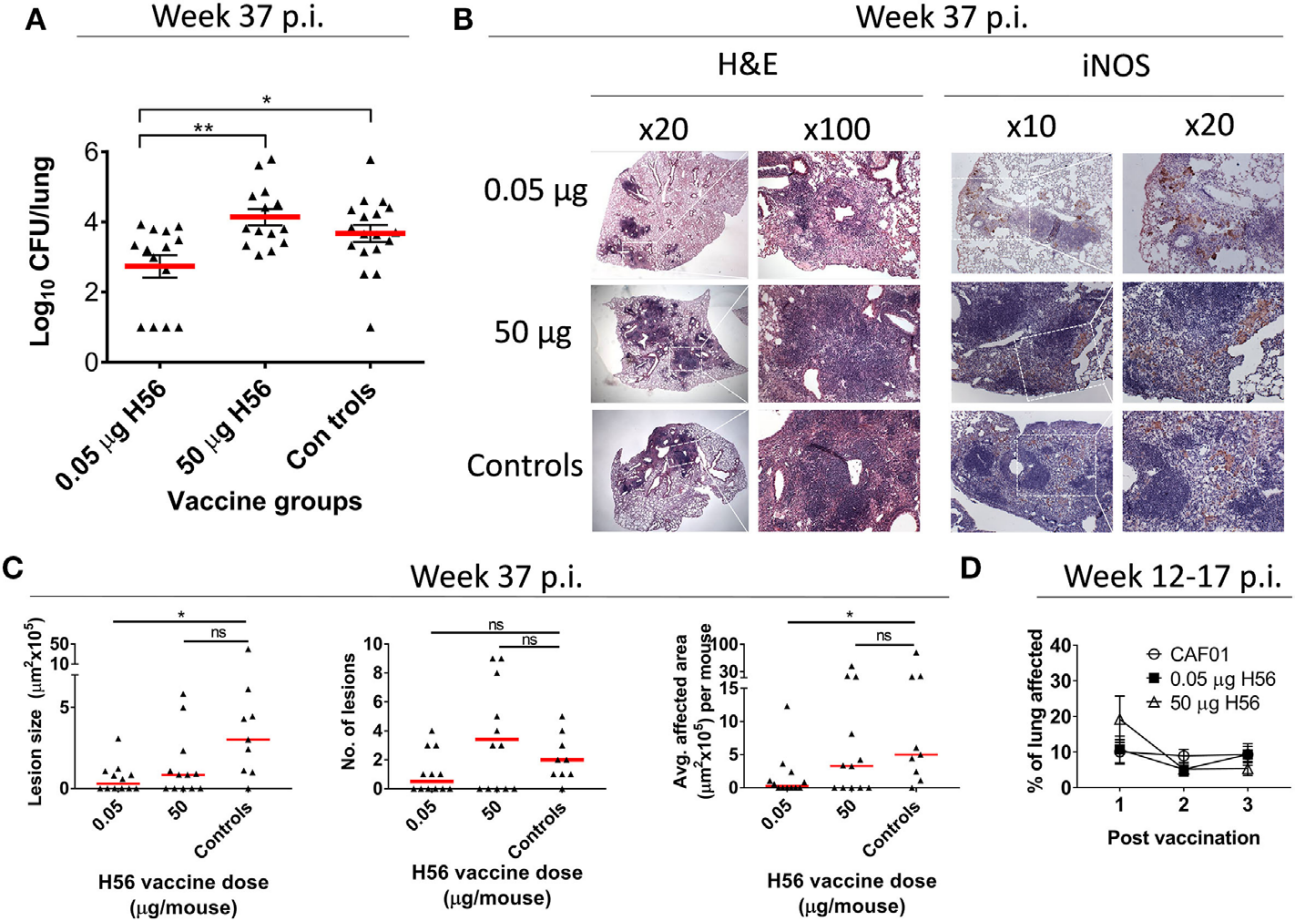
Low dose, but not high dose, H56 vaccination post-exposure reduces bacterial loads and lung pathology. **(A)** lung bacterial loads (log_10_ CFU) 37 weeks after *Mycobacterium tuberculosis* challenge from mice vaccinated with the indicated dose of H56 in the post-exposure models. The histopathology data in **(B,C)** also derives from this experiment (Figure S1B in Supplementary Material, Exp#1). Symbols represent individual mice; lines represent mean ± SEM. **(B)** representative hematoxylin and eosin (H&E) stains (left panels) and inducible nitric oxide synthase (iNOS) immunohistochemistry (IHC; right panels) of lung tissue from the indicated vaccine groups. Regions of higher magnifications are indicated by inserts (dotted white lines) in lower magnification panels, with magnifications (20×, 100×) indicated above panels. **(C)** The overall lesion size, number of lesions, and average area affected by tuberculosis inflammation per section are shown. Symbols represent individual mice; lines represent medians. *p < 0.05 by Kruskall–Wallis with Dunn’s posttest for multiple comparisons. **(D)** extent of lung inflammation 2 weeks after first and 1 week after second and third post-exposure vaccinations (corresponding to 12, 14, and 17 weeks p.i.) as assessed by H&E staining is shown in a separate experiment. Symbols represent mean ± SEM of *n* = 4–5 mice/group. Differences between vaccine groups were assessed using one-way ANOVA and Tukey’s posttest for multiple comparisons. Statistical significant differences are marked by asterisks, *p < 0.05, **p < 0.01. These experiments were performed once each.

Lungs were removed aseptically post mortem at week 37 after aerosol infection with Mtb (25 weeks after end of antibiotic treatment, at time of bacterial load assessment). The right lung cranial lobe of each mouse was fixed by immersion in 10% neutral-buffered formalin and processed for histological examination. Sections were stained using hematoxylin and eosin and were evaluated without prior knowledge of treatment group. Lesions were quantified using computer-aided histomorphometry (Palm^®^robo software, version 1.2.3; Palm Microlaser Technologies AG Ltd., Bernried, Germany) by a pathologist with previous experience of murine models of TB infection (Figure 2). Immunolabelling of iNOS within lesions was carried out following dewaxing and rehydration of sections and an epitope retrieval step where tissue slides were microwaved for 20 min in tri-sodium citrate solution (pH 6.0). Subsequently, treatment of sections with normal goat serum blocking solution (Vectastain ABC kit, Vector Laboratories, Inc., Burlingame, CA, USA) was performed to block endogenous peroxidase. The primary antibody (rabbit polyclonal anti-mouse iNOS/NOSII, Upstate, Lake Placid, NY, USA) was then applied for 1 h at 1/1,000 dilution followed by sequential application of biotinylated goat anti-rabbit IgG (Vector Laboratories) and ABC solution for 30 min, respectively, at room temperature (Vectastain ABC kit, Vector Laboratories). Visualization of target cells followed application of diaminobenzidine-tetrahydrochloride solution (Sigma-Aldrich, Steinheim, Germany) and a hematoxylin counterstain. To evaluate potential vaccine-mediated immune pathology shortly after vaccinations, lungs were removed post mortem 2 weeks after first, and 1 week after second and third (=last) vaccination. The entire left lung lobe of each mouse was fixed by immersion in 10% neutral-buffered formalin and processed for histological examination. The scale of pulmonary inflammation was analyzed using image analysis software (NIS-Elements D 3.0n Nikon Instruments Europe BV, Amstelveen, Netherlands) to determine the percentage lung tissue affected by two TB-pathology specialists (Figure 2D). This experiment only evaluated early pathology and ended 1 week after third vaccination (week 17 post-infection), hence, no protection was measured at week 37 post-infection and the experiment is therefore not referenced in Figure S1 in Supplementary Material.

### Adoptive Transfer Experiment and ESAT-6_4–17_ Tetramer

C57BL/6 mice (*n* = 7/group) were vaccinated three times with a high (50 µg) or lower (5 µg) dose of H56 in CAF01 in a modified post-exposure model, in which mice were infected by the aerosol route with 10–50 CFU of virulent Mtb Erdman. Mice were then subjected to an antibiotic chemotherapy treatment (100 mg/l rifabutin, 100 mg/l isoniazid) in drinking water from week 14–22 of infection. Mice were immunized three times 2 weeks apart starting in the fourth week of chemotherapy (weeks 18, 20, and 22 post challenge; schematic overview in Figure 5A). Spleen and inguinal lymph nodes draining the SOI from post-exposure vaccinated donor mice were isolated 5 days after last vaccination, and CD4 T cells purified by negative CD4 T cell separation (Stemcell kit and magnet). The CD4 T cells were separated to a purity of 91–93%. Enriched CD4 T cells from the high dose animal were then stained with 10 µmol Cell Proliferation Dye 450 (CPD450), and cells from low dose immunized animals stained with 5 µmol CPD670. CD4 T cells pooled from seven high and low dose vaccinated donor mice, respectively, were then pooled in a 1:1 ratio and a total of 1–1.2 × 10^7^ CD4 T cells adoptively transferred into each of seven syngeneic recipient mice infected 4 weeks before with Mtb Erdman (10–50 CFU by aerosol route). 18 –20 h after adoptive transfers, recipient mice were euthanized 3 min after an i.v. injection of anti-CD45.2-FITC. Lung lymphocytes of recipient i.v. stained mice were then subjected to an ESAT-6_4–17_ I-Ab tetramer magnetic bead enrichment (NIH tetramer core facility, Bethesda, MD, USA). To this end, isolated lung lymphocytes were stained with PE-conjugated I-Ab:ESAT-6_4–17_ tetramer at 37°C for 30 min followed by anti PE-bead (Miltenyi, Cologne, Germany) staining at 4°C for an additional 30 min. Tetramer binding cells were then positively enriched by MACS separation on LS columns. ESAT-6 tetramer enriched cells were subsequently surface stained and analyzed for abundance and localization of ESAT-6 specific cells from each donor origin. The CD45.2-i.v. stain allowed for discrimination of vascular and parenchymal cells; CD45.2-stained cells were derived from the lung vasculature, and CD45.2 negative cells derived from the lung parenchyma (protected from the intravascular stain).

### Statistical Methods

Statistical difference in protective efficacy and immunogenicity (total cytokine) of vaccines was evaluated using non-parametric ANOVA (Kruskall–Wallis) and Dunn’s posttest comparing all groups to the controls unless otherwise stated in the figure legends. Normal distribution of bacterial counts was evaluated by D’Agostino and Pearson’s omnibus normality test. Comparisons of boolean cytokine populations between vaccine groups were tested with a two-way ANOVA and Tukey’s posttest comparing all groups against each other. Comparison of functional avidity log_10_(EC_50_)-values was carried out using one-way ANOVA and Tukey’s posttest. IL-2 ratio between high and low vaccine dose was compared by Wilcoxon signed rank test. Differences in the proportion of donor cells homing to the infected lung parenchyma (i.v. −ve) from 50 µg and 5 µg post-exposure immunized mice after transfer into the same recipients were analyzed by a paired Student’s *t*-test. A value of *p* < 0.05 was considered significant. Prism version 7 software (GraphPad) was used for analyses.

## Results

### High Antigen Dose Abrogates Vaccine Protection against TB in the Post-Exposure Setting

In the past decade, our laboratory has focused on understanding and developing efficacious anti-TB vaccines for use in acute, late, and post-exposure stages of Mtb infection. Here we tested the effect of H56 antigen dose on protection in a preventive model, in which mice were vaccinated prior to challenge, as well as in a post-exposure relapse model in which mice are vaccinated with partial antibiotic clearance of bacilli (see Figure S1A in Supplementary Material and Section “Materials and Methods” for model details).

A growing body of evidence from several groups, including our own, has shown the important effects of antigen dose on T cell function and preventive protection against TB (24, 28, 32). To study the effect of vaccine dose on both preventive and post-exposure protective immunity, we retrospectively analyzed data from multiple independent murine experiments conducted in both of these models during the past years in which various H56 doses in CAF01 were included.

Figures 1A,B show typical results of the preventive and post-exposure TB vaccine models with significant protection using the H56 vaccine candidate at a standard dose of 5 µg, in line with previous studies (6, 7). The result of the analysis showed that a wide dose range (0.05–50 µg) of H56 resulted in significant protection levels after preventive vaccination (*p* < 0.0001), whereas a much narrower range of lower doses (0.05–0.5 µg) were protective in the post-exposure model (*p* < 0.01–0.0001; Figure 1C). Importantly, the highest dose (50 µg) completely abolished protection in the post-exposure model and was significantly inferior to post-exposure protection obtained with 0.05 µg H56 [*p* < 0.0001 (significance not shown in graph); Figure 1C]. A meta-analysis format visualizing preventive and post-exposure Δlog_10_-protection with 95% CI of high/low H56 vaccine dose for individual experiments clearly showed the abolished protection in the 50 µg post-exposure group, also at the single experiment level (Figure S1B in Supplementary Material).

Overall, we observed a clear difference between the vaccine dose optimum in the preventive and post-exposure settings, where the highest H56 dose completely abolished protection post-exposure despite giving significant preventive protection. Therefore, from here on, we chose to focus on the most protective low dose of 0.05 µg H56, and the non-protective high dose of 50 µg H56 to further elucidate the dose related difference in post-exposure protection in more detail.

### Only Low Dose Vaccination Reduce TB-Related Pathology in the Post-Exposure Model

In support of the observation that the high H56 dose of 50 µg was unable to restrict pulmonary mycobacterial growth, a histopathological assessment of the lungs at the time of CFU assessment (week 37 p.i.) was performed from one of the experiments included in Figure 1C. The pulmonary bacterial burden from this experiment was in line with the combined data from Figure 1C and showed that a low dose of 0.05 µg H56 given post-exposure was protective, whereas a high dose of 50 µg H56 was not protective (Figure 2A). The histopathological analysis showed that only low dose H56 vaccination resulted in significantly smaller lesions and less total area affected by TB inflammation compared to non-vaccinated controls at the necropsy time point week 37 p.i. (*p* < 0.05; Figures 2B,C). The reduced pathology in the low dose group was not associated with an overall increased macrophage activation as assessed by iNOS staining in lungs of these mice, since comparable levels was detected in the lungs of the three groups at this late time point (Figure 2B). It could be speculated that a high H56 antigen dose given post-exposure potentially could lead to direct immune pathology in the pulmonary lesions due to excess T cell activation. We therefore monitored pulmonary vaccine-related immune pathology shortly (1–2 weeks) after each post-exposure vaccination. At these early time points (week 12–17 p.i.), we found no evidence of increased pathology for any H56 dose compared to non-vaccinated mice suggesting that the difference in the long-term pathological outcome was not the consequence of any immediate exaggerated effect of excessive T cell stimulation post vaccination (Figure 2D). In conclusion, a low dose of 0.05 µg H56 protected mice from TB in the post-exposure model by restricting mycobacterial growth and limiting pulmonary TB-related pathology, and this protection was completely absent after administering a high dose (50 µg) of the same H56 vaccine antigen.

### High Antigen Dose Given Post-Exposure Leads to a More Effector-Driven T Cell Phenotype

**Figure 3:**
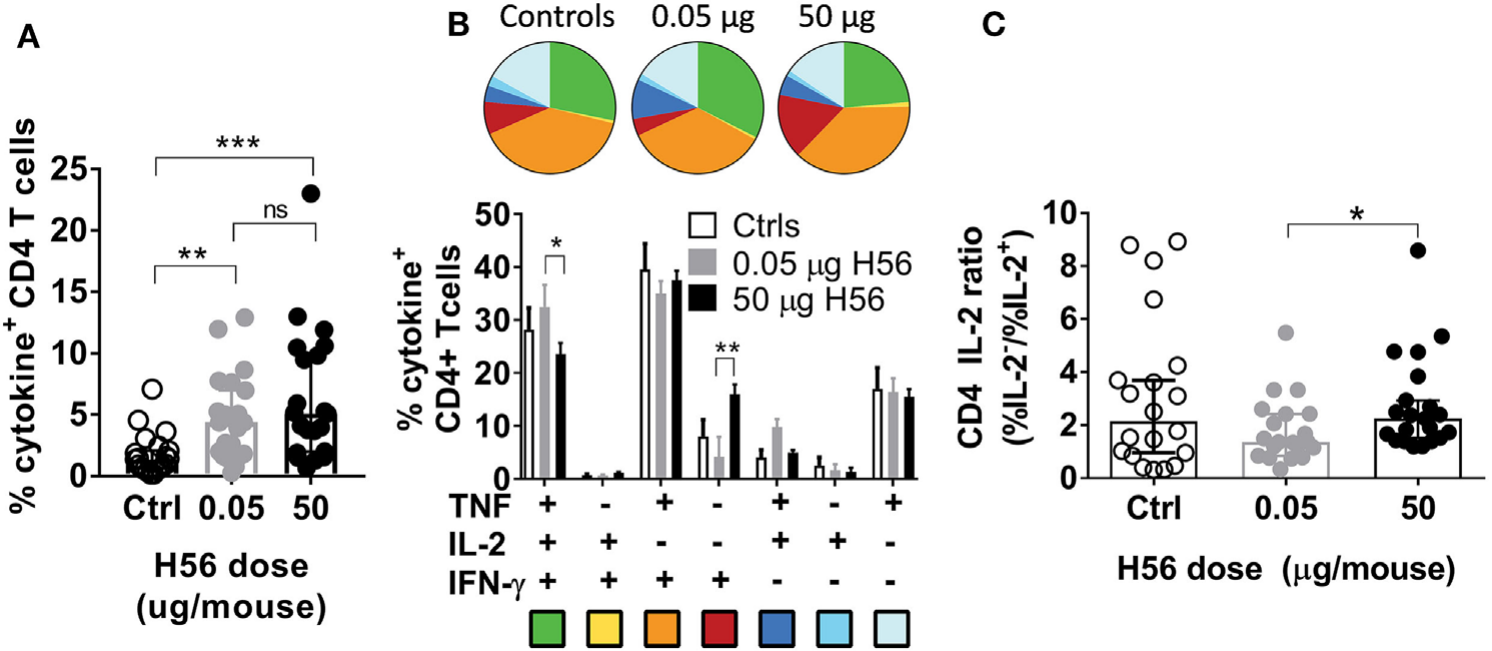
Non-protective, high dose post-exposure vaccination is associated with a more differentiated T cell response. One week post the final vaccination in the post-exposure model, mice were immunized with saline (controls, open bars/symbols), a low (0.05 µg, gray bars/symbols) or high (50 µg, filled black bars/symbols) dose of H56, and were assessed for vaccine-specific immune responses in the lung. **(A)** The proportion of lung CD4 T cells specific for H56 determined by intracellular cytokine stain (producing any cytokine of IFN-γ, TNF, IL-2, or IL-17A) after stimulation *in vitro* with H56 with media control background subtracted. Symbols, individual mice; bar, mean ± 95% CI; **p < 0.01, ***p < 0.001 by one-way ANOVA (Kruskall–Wallis)/Dunn’s posttest for multiple comparisons between all groups. **(B)** Boolean analysis of the same data from panel **(A)**, showing the proportion of lung CD4 T cells producing any combination of the cytokines IFN-γ, TNF, and/or IL-2 after stimulation *in vitro* with H56. Background from media controls was subtracted. Pie charts and bars (mean ± SEM) represent the relative distribution of CD4 T cell subsets producing different cytokine combinations out of total cytokine-producing CD4 T cells (the total for each group equals 100%, relative values were chosen to eliminate variation in response-magnitude between experiments). Pie color-coding is indicated below the bar graph. *p < 0.05, **p < 0.01 by a two-way ANOVA and Tukey’s multiple comparisons (the relative values were normally distributed). **(C)** From the data shown in panel **(B)**, we calculated the relative ability of vaccine specific CD4 T cells to produce IL-2. An IL-2 ratio (%IL-2^−^/%IL-2^+^) was calculated for any CD4 T cell producing IFN-γ, TNF, or IL-2 after H56 stimulation—a higher ratio indicates lower IL-2 production (higher differentiation) of H56-specific T cells. Statistical difference between high and low dose vaccine groups was assessed Wilcoxon’s signed rank test. *p < 0.05. Data combined from seven experiments (see Materials and Methods for experiment inclusion).

Given the significant difference in vaccine efficacy between high and low H56 doses in the post-exposure experiments, we next evaluated the effect of vaccine dose on the T cell response. Multiple experiments were performed with similar outcomes, and we combined the experiments in Figure 1C that contained identical vaccine responses analyzed by ICS from both 0.05 and 50 µg H56 in the same experiment. Combining the Δlog_10_-protection for these five experiments confirmed that 0.05 µg H56 protected significantly better than 50 µg as expected (not shown). We then assessed the magnitude and phenotype of the H56-response in the lungs of mice sacrificed one week after vaccinations (week 17 p.i.). We first observed that high and low dose vaccination induced similar percentages of CD4 T cells producing any of the measured cytokines (IFN-γ, TNF, IL-2, and/or IL-17A) as shown by ICS of H56-stimulated lung lymphocytes (Figure 3A). Hence, the lack of protection in the 50 µg H56 dose group was not related to reduced magnitude of the overall immune response. Second, we performed boolean gating of the three canonical Th1 cytokines, IFN-γ, TNF, and IL-2 in the same dataset as shown in Figure 3A. This showed that high dose vaccination resulted in a subtle, yet consistent, increase in terminally differentiated effector CD4 T cells producing IFN-γ alone (Figure 3B, red pies), whereas low dose H56 vaccination led to more IL-2 producing memory-like T cells also expressing TNF with or without IFN-γ (Figure 3B, green and blue pies, respectively, *p* < 0.013–0.05). Since more differentiated T helper cells lose the ability to secrete IL-2, we calculated and compared the ratio of vaccine specific T cells producing IL-2 from the ICS data shown in Figure 3B. After *in vitro* H56 stimulation, the ratio of cytokine positive CD4 T cells unable to secrete IL-2 (IL-2^−^) to the IL-2-producing (IL-2^+^) CD4 T cells was significantly higher in the high 50 µg H56 dose group compared to the low 0.05 µg H56 dose group (*p* < 0.05; Figure 3C), indicating a greater degree of T cell differentiation in the high dose group.

In conclusion, the high H56 dose of 50 µg led to a similar magnitude of vaccine specific T cells as the low dose; however, the high dose resulted in a tendency toward more differentiated T cells with a lower capacity to produce IL-2.

### High Antigen Dose Given Post-Exposure Leads to a Decrease in T Cell Functional Avidity

**Figure 4:**
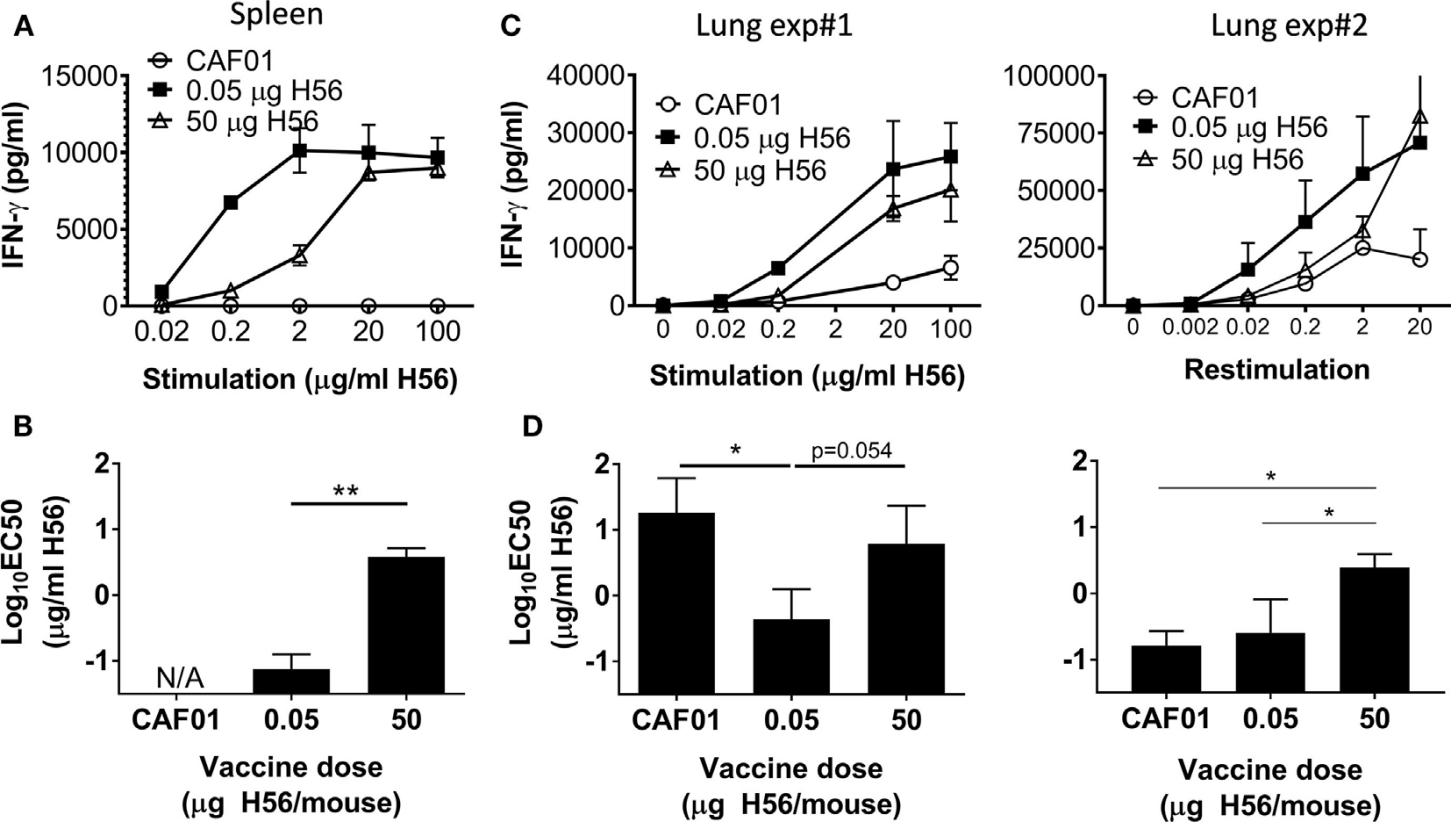
High dose post-exposure H56 vaccination leads to reduced functional avidity. One week post the final vaccination in the post-exposure model, splenocytes **(A)** or lung cells **(C)** from control (open circle) and H56-vaccinated (0.05 µg closed square; 50 µg open triangle) mice were cultured for 3 days with varying concentrations of H56, and IFN-γ release in the culture supernatants was measured by ELISA. **(A)** Data points represent mean ± SD of triplicate cultures of pooled cells from three mice/group (from Exp. 4 in Figure S1B in Supplementary Material). **(B)** EC_50_ was calculated using data from **(A)**; bars, mean ± SD per vaccine group. This experiment was repeated twice. **(C,D)** IFN-γ release and calculated functional avidity (EC_50_) of lung cells from two independent experiments in which subsequent week 37 protection was not assessed. **(C)** Data points represent mean ± SD of triplicate cultures pooled from five mice/group. **(D)** Bars indicate mean ± SD EC_50_ calculated from **(C)**. Statistical differences between functional avidity of vaccine groups were assessed by a one-way ANOVA and Tukey’s posttest for multiple comparisons. *p < 0.05, **p < 0.01. No further identical experiments with lung T cell avidity were performed.

We recently published that low vaccine antigen doses in liposomal CAF adjuvants increased the antigen sensitivity, termed functional avidity, of CD4 T cells (32), and with the very low bacterial loads in the post-exposure model, the ability of T cells to respond to low antigen levels is highly relevant. We therefore compared the functional avidity of vaccine specific CD4 T cells 1 week after high and low dose H56 post-exposure vaccination. Splenocytes from low dose immunized mice responded substantially better to lower concentrations of *in vitro* antigen stimulation compared to the high dose group as reflected by IFN-γ secretion in culture supernatants (Figure 4A). Furthermore, the concentration of antigen required to reach 50% of the maximum response (EC_50_) was significantly higher for the high (50 µg) dose group compared to the low (0.05 µg) dose group (*p* = 0.014; Figure 4B). We next analyzed the functional avidity of H56-specific CD4 T cells in the lungs. In two separate experiments, we observed that low dose vaccination also resulted in H56-specific pulmonary T cells of higher functional avidity compared to high dose vaccination. Thus, *in vitro* stimulation of lung lymphocytes from low dose vaccinated animals resulted in IFN-γ secretion at lower antigen concentrations compared to high (50 µg) dose vaccination (Figure 4C). The antigen concentration needed for 50% maximal activation (EC_50_) of lung lymphocytes was greater after high compared to low dose vaccination (*p* = 0.054 in Exp#1, and *p* = 0.027 in Exp#2; Figure 4D). Moreover, low dose vaccination led to a higher “per cell” IFN-γ production and stronger ESAT-6 tetramer binding, as assessed by flow cytometry in lungs 1 week after vaccination (data not shown), further supporting higher avidity of these vaccine-specific T cells.

In summary, low dose post-exposure vaccination led to protective CD4 T cells with greater functional avidity.

### High Dose Post-Exposure Vaccination Reduces Lung Parenchymal Homing Ability of Vaccine-Specific T Cells

Recent research has shown that a hallmark of protective CD4 T helper cells in murine TB is the ability to home into the lung parenchyma and interact with infected cells, and that this ability is tightly linked to the differentiation state of the T cells (15, 29, 30). Hence, more differentiated T cells are trapped in the lung vasculature and do not enter the parenchyma, where the TB lesions are located. Given that high dose post-exposure vaccination led to increased CD4 T cell differentiation compared to low dose vaccination, we speculated whether the high vaccine dose could potentially also impact the ability of vaccine-primed CD4 T cells to home from the circulation and into the lung parenchyma.

**Figure 5:**
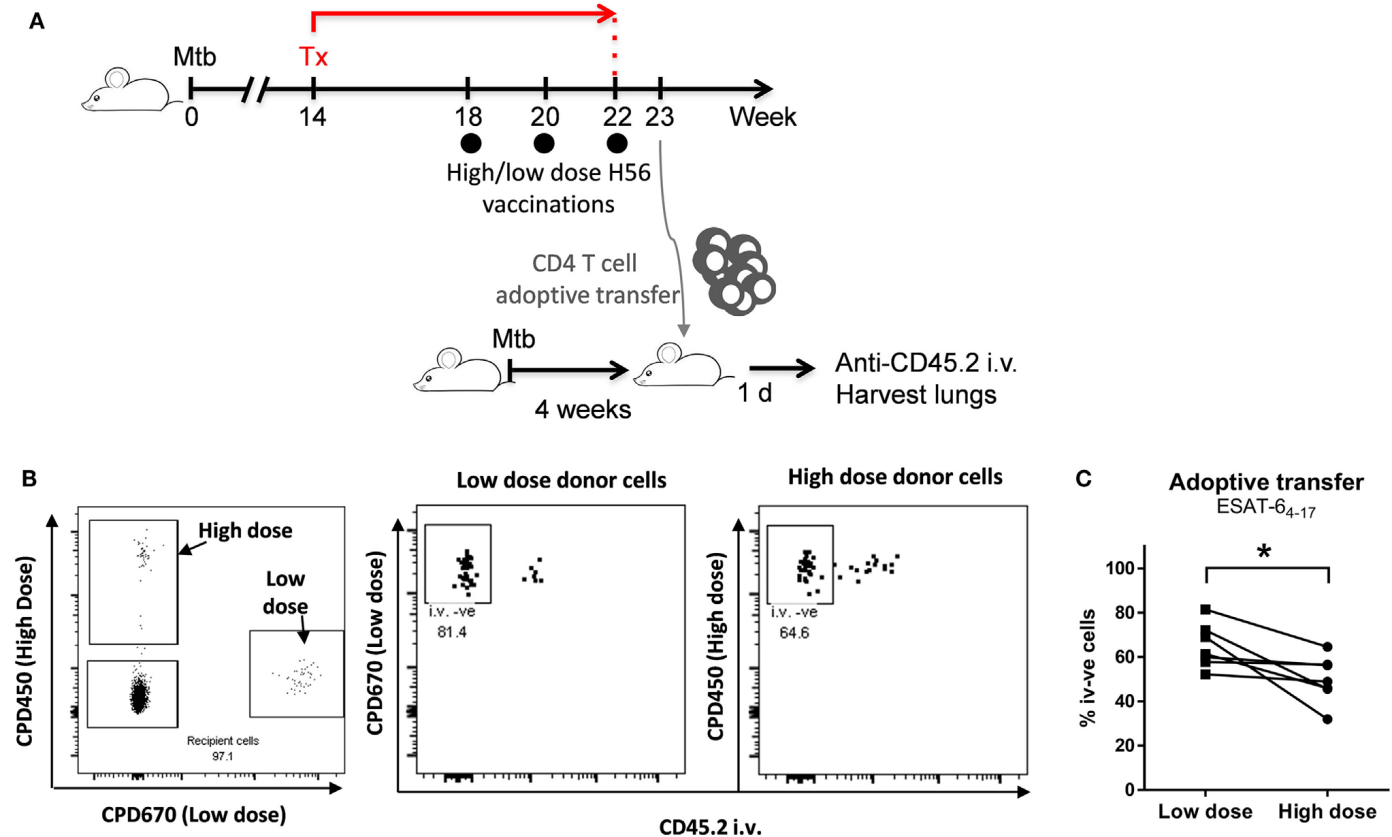
A high H56 dose given post-exposure leads to reduced ability of vaccine-specific CD4 T cells to home into the parenchyma. C57BL/6 mice were vaccinated post-exposure three times with a high (50 µg) or lower (5 µg) dose of H56 in CAF01 in a slightly modified post-exposure model as outlined in **(A)**. Spleen- and dLN-derived CD4 T cells were isolated 5 days after last vaccination. CD4 T cells from high dose vaccinated animals were then stained with Cell Proliferation Dye 450 (CPD450), and cells from low dose vaccinated animals stained with CPD670. Pooled CD4 cells from high and low dose vaccinated animals (*n* = 7) were then combined in a 1:1 ratio and adoptively transferred into seven C57BL/6 mice infected 4 weeks earlier with *Mycobacterium tuberculosis* (Mtb) Erdman (10–50 CFU by the aerosol route). ~20 h after adoptive transfer, recipient mice were euthanized after an i.v. injection of anti-CD45 FITC. I-Ab-ESAT-6_4–17_ tetramer enriched lung lymphocytes of recipient CD45 i.v.-stained mice were then analyzed for abundance and localization of donor cells. The CD45 i.v. stain allowed differentiation of vascular and parenchymal T cells: CD45 i.v.-positive cells derived from the lung vasculature, and CD45 i.v.-negative cells derived from the lung parenchyma (protected from the intravascular CD45 stain). **(B)** The left panel shows a representative dot plot gated on live, I-Ab-ESAT-6_4–17_ + ve lung CD4 T cells (singlets > lymphocytes > live CD4 T cells > ESAT-6 Tet + ve). Double-negative cells, endogenous recipient ESAT-6-specific CD4 T cells. CPD450^+^ cells, ESAT-6-specific CD4 T cells (high dose vaccinated donors). CPD670^+^ cells, ESAT-6-specific CD4 T cells (low dose vaccinated donors). The right plots show representative CD45 i.v. stain of donor cells from low dose (mid panel) and high dose (right panel) vaccinated donors. Percentages represent CD45 i.v. negative cells, i.e., relative proportion of parenchymal CD4 T cells from each donor population. **(C)** Data points represent percentage CD45 i.v. negative (parenchymal) lung CD4 T cells derived from low dose (filled squares) and high dose (filled circles) vaccinated donors gated as shown in **(B)**. Lines represent matched pairs (donor cells transferred into the same recipient). Statistical analysis was performed by a paired Student’s *t*-test. *p < 0.05. This experiment was performed once.

To address this, we co-adoptively transferred donor CD4 T cells purified from mice receiving either low or high post-exposure vaccine doses into Mtb-infected syngeneic recipient mice (Figure 5A). We used ESAT-6:MHC-II tetramers combined with intravital i.v. staining to subsequently track the lung homing capacity of the transferred donor cells. CD4 T cells were isolated by magnetic enrichment (negative selection) from spleens and inguinal lymph nodes (draining the SOI) from mice post-exposure vaccinated with either a high or a low H56 dose. For the high dose, we used 50 µg H56, while for the low dose we chose 5 µg H56, since we have observed more variation in the magnitude of vaccine-specific T cells using 0.05 µg, and observed similar T cell differentiation after 0.05 and 5 µg H56. After CD4 T cell enrichment, donor cells from high and low dose post-exposure vaccinations were differentially stained with cell-tracking dyes in order to distinguish donor cells after co-adoptive transfer into the same infected mouse (Figure 5A). Roughly 20 h after transfer, the ability of I-Ab:ESAT-6_4–17_ specific donor cells to home into the lung parenchyma was analyzed by magnetic enrichment of ESAT-6 tetramer binding cells combined with an intravascular staining technique, where fluorescent anti-CD45.2 administered intravenously (i.v.) prior to euthanasia, allowed separation of cells from the vascular (CD45.2 i.v.^+^) and parenchymal (CD45.2 i.v.^−^) lung compartments. ESAT-6-specific donor cells from high and low dose post-exposure vaccinated animals could clearly be distinguished from each other, and from the double negative endogenous recipient lung cells (Figure 5B, left panel). Importantly, donor cells deriving from low dose vaccinated animals showed an improved ability to home into the lung parenchyma of infected recipients, as seen by the increased percentage of CD45.2 i.v. negative cells in lungs of recipient mice (Figure 5B, middle vs. right panel). Moreover, a significantly higher proportion of donor cells from low dose post-exposure vaccinated mice could be observed within the lung parenchyma (CD45.2 i.v. negative) compared to donor cells from high dose vaccinated mice after transfer into the same recipient mice (*p* = 0.026; Figure 5C), thus directly demonstrating an improved lung parenchymal homing capacity. In conclusion, the high dose of 50 µg H56 led to a decrease in the ability of vaccine-specific T cells to home into the lung parenchyma associated with highly differentiated cells, which could potentially be a contributing factor in the loss of protection observed from high H56 dose post-exposure vaccination.

## Discussion

In this study, we observed that a wide range of high and low vaccine doses were protective in a preventive murine TB model, but similar high vaccine doses were detrimental to post-exposure vaccine protection. Loss of protection after high dose vaccination was associated with a more effector-driven phenotype and decreased functional avidity, which further correlated with a decreased parenchymal homing ability of the vaccine specific T cells.

TCR-stimulation strength has been linked to the type of response since early studies performed in the 1980s and 1990s (33–35), showing the strength of stimuli could regulate Th1/2 polarization, and later also to play a role in induction of follicular helper T cells (36, 37), regulatory T cells [reviewed in Ref. (38)], as well as memory induction (39). The importance of vaccine antigen dosing and subsequent protection has been observed in a number of infectious diseases and cancers in animals and humans, with the common conclusion that higher doses lead to increased immune responses and improved protection (40–44). However, high antigen concentrations can accelerate T cell differentiation (45), and our data clearly show the importance of carefully titrating vaccine antigen dose, not only for a specific disease, but also for different stages of that specific disease to obtain optimal protection. Importantly, while a broad range of H56 vaccine antigen doses were protective in prophylactic vaccination, only a narrow range of lower doses were protective in post-exposure vaccination. The wide protective range (saturating at 10^3^-fold increase from lowest protective dose) of H56/CAF01 given preventively is in contrast to our previous observations using a similar vaccine and adjuvant, H4 (Ag85B-TB10.4) in IC31, which had a narrower range of both protection and immunogenicity (0.05–1 µg). Importantly, in the H4/IC31 study, protection correlated closely with the magnitude of vaccine response that decreased dramatically at doses higher than 1 µg of H4. This underlines the influence of the adjuvant system as very similar vaccine molecules given in the CAF01 vs. IC31 result in different immune responses and optimal doses, with CAF01 having a broader plateau for maximum responses than IC31 that sharply decline at all doses above 1 µg (46). The different H56-performance pre/post-exposure is consistent with several reports showing that effective preventive TB-vaccine candidates did not protect when given therapeutically, and therapeutic vaccination even aggravated disease in some cases (4, 5). The lower optimal protective vaccine dose in the post-exposure setting could reflect strong Mtb-priming of T cells to vaccine antigens, in turn leading to greater sensitivity of those T cells to overstimulation after vaccination.

Overstimulation after high dose vaccination could be particularly important with vaccines containing ESAT-6, since recent work in humans showed that CD4 T cells recognizing ESAT-6 are more sensitive to exhaustion due to the high pulmonary expression of this antigen compared to less highly expressed antigens such as Ag85B (18). In line with this, the ESAT-6 antigen itself has been shown *in vitro* to hold immune-regulatory properties, both anti-inflammatory (reduction of macrophage IL-12 release and T cell activation) as well as proinflammatory (macrophage IL-6 production and lung epithelial IL-8 production) as well as impacting the Th1/Th17 balance (47–49). Thus, high concentrations of ESAT-6 in a vaccine could potentially increase these effects. However, as ESAT-6 in the H56 molecule is flanked by Ag85B and Rv2660c on either side, it is unknown whether the H56-contained ESAT-6 exhibits any of these biological functions.

Although our study does not pinpoint one particular T cell deficiency as responsible for the lack of protective effect, it is striking that the high dose vaccine response have an overall impaired T cell quality as evidenced by reduced functional avidity, increased terminal T cell differentiation, and impaired ability to home into the infectious site in the parenchyma. The lower ability to home from the vasculature into the infected lung parenchyma may be the sole consequence of the more differentiated state of the T cells as suggested by recent studies (16, 29, 30). However, it may also relate to the lower functional avidity of the T cells that render them less sensitive to minute concentration of antigens in the infected sites. Hence, we suggest that low dose vaccination given post-exposure is sufficient to drive a protective immune response, whereas higher doses negatively impacts T cell quality and protective capacity. These results are highly relevant for clinical vaccine studies involving QFT+ individuals and suggest that antigen doses must be carefully investigated in clinical trials targeting different populations.

## Ethics Statement

This study was carried out in accordance with the recommendations and regulations of the Danish Ministry of Justice and animal protection committees by Danish Animal Experiments Inspectorate Permit 2009/561-1655, 2012-15-2934-00272, 2014-15-2934-01065, and in compliance with EU Directive 2010/63 and the U.S. Association for Laboratory Animal Care recommendations for the care and use of laboratory animals. Protocols were approved by the SSI ACUC headed by DVM Kristin Engelhart.

## Author Contributions

RB, TL, JW, EA, and PA conceived and designed the studies. RB, TL, and JW performed murine TB experiments and analyzed the data. CV, P-JC, and JC performed histopathological analysis. RB, RM, and PA drafted the manuscript. RB, TL, JW, EA, PA, and RM finalized the manuscript.

## Conflict of Interest Statement

PA and EA are co-inventors of patents regarding the use of H56 [#WO0011214 (Molecular differences between species of the *M. tuberculosis* complex); PA and EA, #WO2006136162 (Tuberculosis vaccines comprising antigens expressed during the latent infection phase); PA] and CAF01 [#WO0069458 (Adjuvant combinations for immunization composition and vaccines); PA]. All rights have been assigned to the SSI, a state owned not-for-profit research organization, and the authors’ co-inventorship did not influence design of studies or preparation of the manuscript. There are no further patents, products in development, or marketed products to declare.
