# Supplementary material for: High Antigen Dose Is Detrimental to Post-Exposure Vaccine Protection against Tuberculosis
Source: Front Immunol. 2018 Jan 15;8:1973. doi: 10.3389/fimmu.2017.01973 (PMC5775287; doi:10.3389/fimmu.2017.01973)
Supplement: Supplementary file 1 [file Data_Sheet_1.PDF]

Supplementary Material

High antigen dose is detrimental to post-exposure vaccine protection against tuberculosis

Rolf Billeskov[1*§], Thomas Lindenstrøm[1§], Joshua Woodworth[1], Cristina Vilaplana[2], Pere-Joan Cardona[2], Joseph P. Cassidy[3], Rasmus Mortensen[1], Else Marie Agger[1], Peter Andersen[1*].

[1]Department of Infectious Disease Immunology, Statens Serum Institut, Copenhagen, Denmark. [2]Unitat de Tuberculosi Experimental, Institut per a la Investigació en Ciències de la Salut Germans Trias i Pujol. CIBER Enfermedades Respiratorias. Universitat Autònoma de Barcelona. Badalona, Spain. [3]Veterinary Sciences Centre, School of Veterinary Medicine, University College Dublin, Belfield, Dublin, Ireland.
[§]These authors contributed equally to this work

***Correspondence:**

Rolf Billeskov, rolf_bs@yahoo.com, Peter Andersen, pa@ssi.dk

Keywords: tuberculosis, post-exposure vaccination, vaccine dose, T cell quality, functional avidity, caF01, h56, adjuvant

Running Title: Low-dose vaccination protects against LTBI

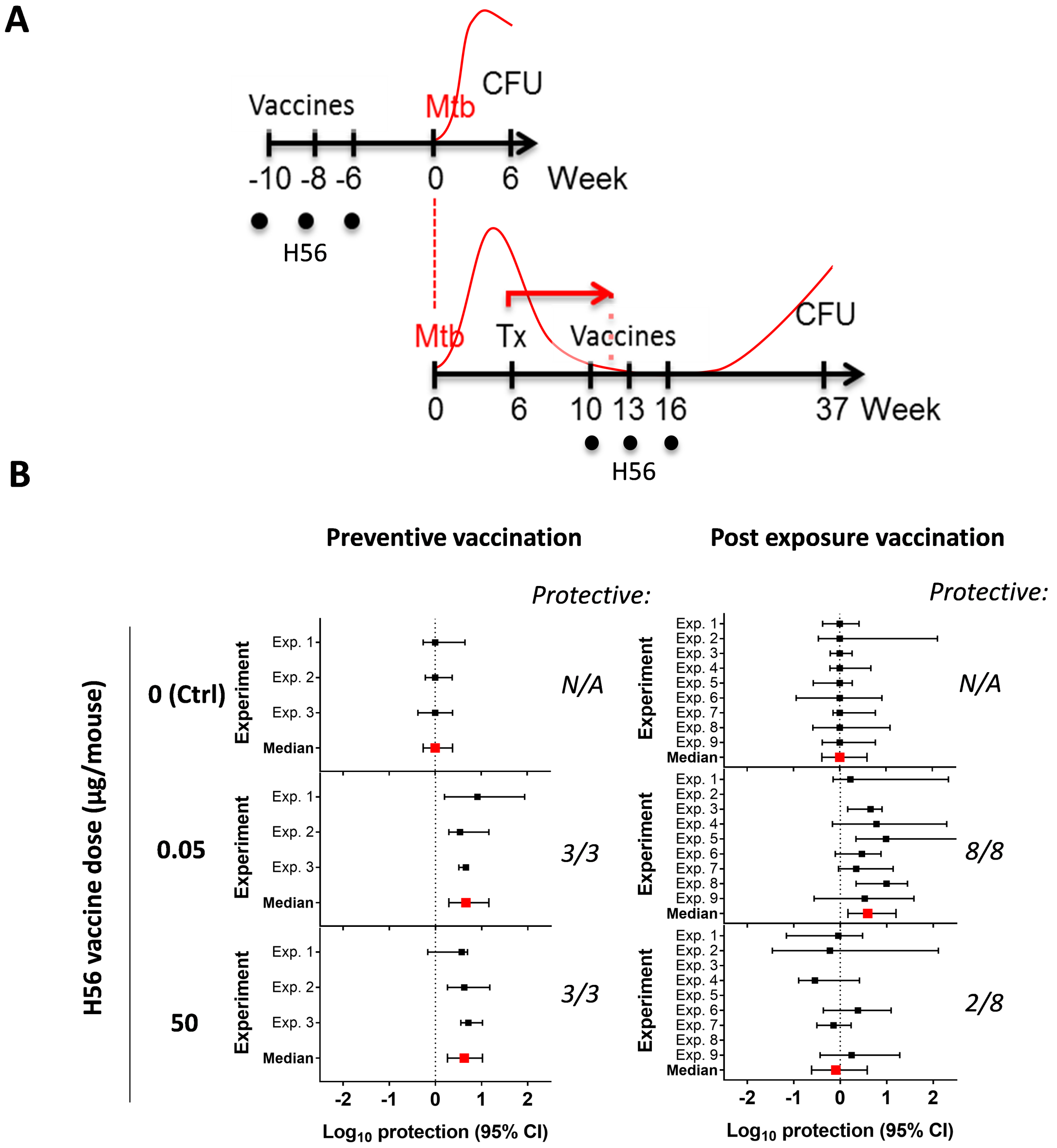

Supplementary Figure S1. Low H56 dose is required for post exposure, but not preventive, vaccine-mediated protection against TB

A, schematic overview of the preventive and post exposure TB vaccine models used. See MM for details. Filled circles illustrates timing of vaccinations, red curves illustrates bacterial loads (arbitrary scale). B, in order to visualize the protection of different doses, but also the considerable variation between experiments observed in the post exposure model, data was set up in meta-analysis format. CB6F1 mice were immunized s.c. according to the preventive or post exposure model schedule outlined in panel A with either 0.05 or 50 µg H56 in CAF01 or CAF01 alone as a control, and protection assessed in lungs either 6 (preventive) or 37 (post exposure) weeks after challenge. Black symbols represent median $\Delta\log_{10}$-protection with 95% CIs of the median of individual experiments indicated on the Y-axis, while the red symbol is the median of all the performed experiments. The proportion of experiments in which the vaccine resulted in protection (defined here as any positive $\Delta\log_{10}$-protection value) is indicated by fractions. Experiment numbers (*e.g.* Exp. 1) refers to the same experiment for each dose within each model (preventive and post-exposure).
